# Being mindful of our insomnia can get us to sleep? - Mindfulness aproach to sleep disorders

**DOI:** 10.1192/j.eurpsy.2023.2347

**Published:** 2023-07-19

**Authors:** R. B. Cohen, I. M. Pereira, M. G. Marguilho, M. C. Sousa, B. V. Ferreira

**Affiliations:** 1 Clínica 5; 2 Clínica 4, Centro Hospitalar Psiquiátrico de Lisboa, Lisboa, Portugal

## Abstract

**Introduction:**

Sleep disorders (e.g., insomnia) are extremely prevalent in our population and are intimately associated with distress and productivity impairment. It is estimated that between 40 to 60% of people suffering from a sleep disorder have an underlying psychiatric diagnosis.

Mindfulness, which is described as the quality or state of being self-conscious or aware of something, has shown to be a potential helpful therapy in insomnia.

**Objectives:**

Therefore, and due to the lack of new and effective treatment approaches, we did a non-systematic review of the positive impact of mindfulness in quality of sleep.

**Methods:**

Bibliographic research through PubMed, Web of Science and Springer Link.

**Results:**

The mindfulness tools that may be linked to its therapeutic effects include the awareness state and conscious posture to respond when perceiving insomnia symptoms, as well as the modulation of sleep-related arousal courses. These can be primary when directly related to the inability to sleep, or secondary if considering the relationship with thoughts about sleep (such as the tendency to create bias in the attention and perception of sleep related thoughts).

Formerly, mindfulness-based cognitive therapy (MTPC) was designed for the treatment of chronic depression and has shown to be efficacious. It was hypothesized that interoceptive dysfunction in the insula, commonly observed in anxiety and depression, may respond to MTPC by the gained interoceptive awareness, which provides advantage to adapt to life challenges and ongoing adjustments.

**Conclusions:**

Based on the currently available literature, mindfulness-based strategies may be a valuable treatment option in sleep disorders, especially for patients with concomitant mental illness. Therefore, it is necessary further research to standardize in terms of type of approach, duration, and outcome measures since it seems promising as an intervention for insomnia.

**Disclosure of Interest:**

None Declared

